# The Evidence-based Practice Attitude Scale-36 (EBPAS-36): a brief and pragmatic measure of attitudes to evidence-based practice validated in US and Norwegian samples

**DOI:** 10.1186/s13012-017-0573-0

**Published:** 2017-04-04

**Authors:** Marte Rye, Elisa M. Torres, Oddgeir Friborg, Ingunn Skre, Gregory A. Aarons

**Affiliations:** 1 Department of Psychology, Faculty of Health Sciences, UiT The Arctic University of Norway, N-9037 Tromsø, Norway; 2 Division of Mental Health and Substance Abuse, University Hospital of North Norway, Tromsø, Norway; 3 Department of Psychiatry, University of California, San Diego, CA USA

**Keywords:** Evidence-based practice, Evidence-based practice in psychology, Evidence-based treatments, Interventions, Implementation, Attitudes, Therapists, Mental health

## Abstract

**Background:**

Short and valid instruments for measuring factors facilitating or hindering implementation efforts are called for. This article describes (1) the adaptation of a shorter version of the Evidence-based Practice Attitude Scale (EBPAS-50 items), and (2) the psychometric properties of the shortened version in both US and Norwegian data.

**Methods:**

The US participants were mental health service providers (*N* = 418) recruited from clinics providing mental health services in San Diego County, California. The Norwegian participants were psychologists, psychiatric nurses, and psychology students (*N* = 838) recruited from the Norwegian Psychological Association and the Norwegian Nurses Organization. A confirmatory factor analysis (CFA) approach was used.

**Results:**

The reduction resulted in 36 items named EBPAS-36, and the original 12 factor model was maintained. The EBPAS-36 had acceptable model fit, as indicated by a low degree of misspecification errors in both the US (RMSEA = .045 (CI_90%_ .040–.049); SRMR = .05) and the Norwegian data (RMSEA = .052 (CI_90%_ .047–.056, SRMR = .07). Incremental model fit was fair in the US (CFI = .93, TLI = .91) and in the Norwegian samples (CFI = .91, TLI = .89). The internal consistency (Cronbach’s α) in the US and the Norwegian samples were good for the total EBPAS-36 score (.79 and .86, respectively) and were ranged from adequate to excellent for the subscales (US .60–.91 and Norway .61–.92).

**Conclusions:**

The EBPAS-36 has adequate psychometric properties both in US and Norwegian samples, hence indicating cross-cultural validity. It is a brief, pragmatic, and more user-friendly instrument than the EBPAS-50, yet maintains a broad scope by retaining the original 12 measurement domains.

**Electronic supplementary material:**

The online version of this article (doi:10.1186/s13012-017-0573-0) contains supplementary material, which is available to authorized users.

## Background

Most evidence-based interventions never become implemented in real-world practice despite a substantial focus on implementation of evidence-based psychological interventions [1, 2]. A remedy is to increase knowledge about what makes implementation successful, and hence development and validation of pragmatic, yet psychometrically strong instruments, becomes crucial [3]. Use of instruments that cover a broad area of factors that facilitate or hinder implementation may provide valuable knowledge to help tailor implementation strategies in order to overcome implementation obstacles. In contrast, the use of poor quality instruments might slow advances of the implementation knowledge base [3, 4], which ultimately may negatively influence the quality of services provided.

Several concerns regarding instrumentation may impede advances in implementation science. This includes a growing number of frameworks, theories or models, an increasing diversity in the operationalization of constructs, improper psychometric testing of instruments, and scant practicality and pragmatism of the available instruments [3–9]. The Society for Implementation Research Collaboration (SIRC) Instrument Review Project [3] recently identified over 420 instruments covering 48 different implementation constructs: “factors inside domains (characteristics of the intervention, characteristics of individuals involved in the implementation, outer settings, inner setting, process, implementation outcomes and client outcomes) that may predict, moderate, or mediate evidence-based intervention dissemination and implementation, as well as implementation outcomes” (p. 3). The preliminary results from the SIRC project suggest that few instruments are psychometrically strong or have been developed through a sufficiently systematic approach [3].

Moreover, the implementation process encompasses different phases involving complex multilevel challenges, such as the exploration, the preparation, the implementation, and the sustainment phase characterized by the Exploration Preparation Implementation and Sustainment (EPIS) implementation framework [10]. In all phases, specific characteristics of the clinical interventions, the patients, the health care professionals, the organizations, and even the policies of health authorities may involve barriers or facilitators for a successful implementation [5, 11–17]. Regarding the health care professionals, one factor that affects the process and outcomes of implementation is service providers’ attitudes to evidence-based practice (EBP) [11, 13, 18, 19]. This is important as it may influence the initial process of deciding whether to launch new practices, the actual implementation process, and how to sustain of interventions efforts within service settings [11, 13].

The Evidence-based Practice Attitude Scale (EBPAS) was developed from theories of dissemination and implementation in mental health, as well as consultations with mental health service providers and researchers [11, 13]. The original EBPAS consisted of 15 items (EBPAS-15) covering four attitude domains: (1) the intuitive *appeal* of EBP, (2) the likelihood of adopting EBP given *requirements* to do so, (3) *Openness* to new practices, and (4) the perceived *divergence* of one’s usual practice with research-based/academically developed interventions. The EBPAS-15 has good psychometric properties [11, 20–23] and is highlighted as psychometrically strong by the SIRC Instrument Review Project [3]. The scores from the EBPAS-15 are associated with relevant provider demographic characteristics, organizational characteristics, leadership [11, 12, 20, 23], as well as provider adoption and use of EBP [24]. For instance, higher educational status is associated with more favorable attitudes [11]. Also, higher levels of positive leadership styles [20] and more constructive organizational culture [23] is associated with more positive provider attitudes, while poorer organizational climate is associated with greater perceived divergence between ones usual practice and EBPs [23]. More recent work has expanded the purview of attitudes and resulted in the development of eight additional domains dispersed across 35 new items (EBPAS-50) [13]: (5) the *limitations* of EBPs, (6) the EBPs *fit* with values and needs of client and clinician, (7) the negative perceptions of *monitoring*, (8) the *balance* between perceptions of clinical skills and science as important in service provision, (9) the time and administrative *burden* with learning EBPs, (10) *job security* related to expertise in EBP, (11) perceived *organizational support*, and (12) positive perceptions of receiving *feedback*.

The expansion from 15 to 50 items covers a wider domain of attitude concepts. However, cross-cultural translations and validation studies are lacking. As part of a Norwegian survey among psychologists and nurses examining challenges with implementation of evidence-based interventions and systems for improving the quality of mental health service settings, a Norwegian translation of the EBPAS-50 was included. Given the need for briefer, yet reliable and valid instruments [3–6], we examined ways of shortening the EBPAS-50.

### Aims of the present study

The present study aimed to shorten the original EBPAS-50 but maintain the original 12 subscales. Furthermore, we examined the factor structure, and the reliability of its subscale scores across two cultures contexts (US and Norway). We expected that the shortened version would have higher user acceptability, retain the original factors structure, show satisfactory reliability, and display indices of good convergent and discriminant validity.

## Methods

### US: procedure

The study was approved by the appropriate institutional review boards prior to recruitment, and informed consent was obtained prior to administering surveys. The research team recruited participants from mental health clinics providing mental health services for children, adolescents, and families in San Diego County, California, United States. Of the initial 99 county run and contracted programs identified, 72 programs were eligible because they provided either outpatient or day treatment mental health services. Twenty-six of the 99 clinics were identified as ineligible because they were residential treatment facilities, lacked the appropriate organizational structure (i.e., no supervisor or program manager for the clinic), or due to inability to make contact with the program. Of the 72 eligible programs, seven programs refused (90.3% response rate). The total number of eligible participants from the 65 participating programs was 440, of which 435 agreed to participate (98.9% response rate). Fifteen individuals were administrative assistants and were not asked to respond to the EBPAS portion of the survey, and two individuals were excluded due to missing data resulting in a total sample size of 418.

### Norway: procedure

Participants were invited by emails sent out by the Norwegian Psychological Association to half of their members (Norwegian sample 1, *N* = 3598) and by the Norwegian Nurses Organization, professional group for nurses in mental health and substance abuse, to all of their members (Norwegian sample 2, *N* = 1436). The survey was also announced on the Internet sites of these two organizations. The invitation email contained a web link providing access to the survey, as well as information about the study provided by the research group. All respondents provided informed consent according to recommendations of the Norwegian data protection authority for the project. Completion of the survey was accepted as consent to participate in the project. The online SurveyMonkey software was used to collect data during May–October 2014. One and two reminders were sent to samples 2 and 1, respectively. For incentives, all participants had the opportunity to participate in random drawings for one iPad mini, and two psychology and nursing handbooks.

A total of 856 psychologists and psychology students (24.0% response rate for sample (1) and 191 nurses (13.3% response rate for sample (2) completed the survey (*N* = 1047). Subjects not completing any of the EBPAS-50 items were excluded, as well as those with missing data for whole subscales, >1 item on a 3-item scale or >2 items on a 4-item scale (*N* = 209). Thus, the final Norwegian sample included data from 838 respondents.

### Norwegian translation procedure

The Norwegian translation of the EBPAS-50 was conducted by the first author (MR) in 2013, and back-translated by a professional. The procedure followed recommended guidelines for cross-cultural translation, adaptation, and validation of instruments [25]. Any deviations between the original and the back-translated version were resolved by a consensus discussion between the first (MR) and the last author (IS). The final Norwegian version was reviewed, revised, and approved through an iterative process that resulted in consensus between MR and the original EBPAS-50 author (GAA). The measure was then given to a sample of clinicians, psychology and PhD students, and comments related to readability were used to finalize the translation.

Some translational adaptions with regard to the definition of the concept of EBP were made for the written instructions of the instruments. In the original version, the instructions specified that “evidence-based practice” refers to any intervention that is supported by empirical research. The Norwegian instructions were limited to evidence-based interventions (i.e., therapies, methods). This was consistent with the American Psychological Association and Norwegian Psychological Associations’ definitions of evidence-based psychological practice. It thus makes an important distinction between the more comprehensive concept of *evidence-based practice*, referring to the integration of the best available research with clinical expertise in the context of patient characteristics, culture, and preferences, and *evidence-based treatments and interventions*, referring to specific research-supported interventions [26, 27].

### Measures/assessment

#### US: demographic characteristics

Participants’ gender, age, ethnicity/race, level of education, primary discipline, years worked in mental health, and years worked in current agency were collected.

#### Norway: demographic characteristics

Participants’ gender, age (response categories < 30 years, 31–40 years, 41–50 years, 51–60 years, and > 60 years), level of education, profession, and years worked in substance abuse- and/or mental health service were collected.

#### The Evidence-based Practice Attitude Scale (EBPAS-50)

EBPAS-50 is a 50-item instrument developed to assess mental health and social service providers’ attitudes toward adopting EBP [13]. The 50 EBPAS-items cover 12 subscales: appeal (four items), requirements (three items), openness (four items), divergence (four items), limitations (seven items), fit (seven items), monitoring (four items), balance (four items), burden (four items), job security (three items), organizational support (three items), and feedback (three items). The 12 subdomains sum up in a higher order total scale score representing respondent’s global attitudes toward evidence-based practice. The items are formulated as statements, and responses are given on a 5-point Likert scale ranging from 0-“not at all” to 4-“to a very great extent”. In order to assess different perspectives and to reduce response biases, 23 items belonging to five subscales (divergence, limitations, monitoring, balance, and burden) are negatively framed. According to the EBPAS-50 scoring instructions, for the total score these items are reversed scored, and the mean subscale scores recomputed, before a mean score for the total EBPAS-50 item score is computed. A higher total score indicate a more positive attitude towards adoption of evidence-based practice.

#### Open-comment fields

For the Norwegian survey, respondents also had the opportunity to convey their opinions and to supply supplementary information in open-comment fields to provide feedback regarding content and the measure overall.

### Statistical analyses

Confirmatory factor analyses (CFA) for item reduction evaluations were conducted in Mplus v7.2. The model specification was based on the 12 subscales of the expanded EBPAS-50. The Norwegian sample was split using the first sample to identify a shorter version (*N* = 413), and the second sample to validate the factor structure (*N* = 425). For the US CFA’s, the same sample was used for both the reduction and the validation process. The parameters were estimated with the full information maximum likelihood procedure (FIML), and robust standard errors (MLR) were requested in order to accommodate for non-normal item distributions. The MLR procedure is efficient and works comparably well as weighted least squares procedures for ordinal data with five or more ordinal categories [28]. The following model fit indices were used: χ^2^, root mean square error of approximation (RMSEA), standardized root mean error (SRMR), and comparative fit indices (CFI) [29]. Following Hu and Bentler’s cutoff recommendations [30], RMSEA values close to .06, SRMR close to .08, and CFI close to 0.95 indicate acceptable model fit. The US sample controlled for the nested data structure of providers within program. Given the aim to reduce the length of the EBPAS-50 while retaining the original 12 factors, a minimum of three items per factor were retained. Subscales containing four or more items were thus shortened based on a combination of the following criteria: (1) retain items with the highest factor loadings; (2) evaluations of modification indices; (3) items being conceptually similar or adding unique information. The reduction process was done separately for the US and the Norwegian samples, and the resulting versions were compared and discussed before reaching a final consensus version. It is important to note that our primary goal was to develop a brief and a pragmatic measure that represented the original constructs identified in the EBPAS-50 subscales. The removal procedure put weight on keeping items that preserved the content and meaning of the subscales, in addition to information about factor loadings and scale reliability.

### Calculations of test parameters

The validity of the questionnaire was measured by acceptability, the percentage of items left unanswered, and the interpretability of the components. SPSS version 22 was used for basic statistical analyses and estimation of internal consistency (Cronbach’s α).

## Results

### Samples

For the US sample, the average age of the 418 participants was 36.3 (SD = 10.6; range = 21–66), and the majority of respondents were female (79.8%). Participants worked in the mental health services field for a range of 0–43 years, in child and/or adolescent mental health services for a range of 0–42.7 years, and in their present agency for a range of 0–29.1 years participants’ areas of primary discipline included: 2.5% child development, 0.2% drug/alcohol counseling, 1.5% human relations, 48% marriage and family therapy, 1% nursing, 0.2% probation, 0.5% psychiatry, 15.3% psychology, 24.6% social work, and 6.2% other discipline. Participant demographic characteristics for the US sample are provided in Table 1.

**Table 1:** Demographic characteristics of the US sample

Characteristics	Values
Gender	
Female	79.8%
Male	20.2%
Race/ethnicity	
Caucasian	54%
African American	6.7%
Hispanic	23%
Asian American	4.2%
Native American	0.2%
Other	11.9%
Highest education	
Ph.D./M.D.	6.9%
Master’s degree	58.6%
Some graduate work	5.7%
Bachelor’s degree	12.3%
Some college	2.2%
Associate’s degree	1.7%
High school diploma	0.5%
Less than high school diploma	0.2%
Age	
Mean (SD)	36.3 (10.6)
Tenure in mental health (years)	
Mean (SD)	8.5 (7.7)
Tenure in child/adolescent mental health (years)	
Mean (SD)	7.5 (7.6)
Tenure with agency (years)	
Mean (SD)	3.1 (4.2)

For the Norwegian sample, the majority of the sample (*N* = 838) were in the age category 31–50 years, one third were older than 50, and a sixth younger than 30. An estimated mean age for the Norwegian sample was thus 43,4 years, based on the midpoint of each age category and weighted by the numbers falling in each category (for those in the category under 30, a midpoint of 27 years was chosen, since psychologists and nurses graduate at minimum age 24–25. The majority of the participants were female (68.6%). Clinical psychologist reported working in the substance abuse and mental health service field for a range of 0–45 years, while nurses reported a range of 2–42 years of experience. Sixty-two (7.4%) participants were psychology students following a six-year university education and training program in clinical psychology leading to the postgraduate cand. psychol. degree, 655 were authorized clinical psychologists with an accomplished postgraduate cand. psychol. degree (78.1%), and 121 were authorized nurses with an accomplished Bachelor’s degree in nursing (14.5%). Participants came from a diverse setting of mental health services, with the largest group representing outpatient units for adults (18.0%). Participant demographic characteristics for the Norwegian sample are presented in Table 2.

**Table 2:** Demographic characteristics of the Norwegian sample (*N* = 838)

Characteristics	Values
Gender^a^	
Female	68.6%
Male	31.4%
Highest education clinical psychologists^b^	
Both Ph.D and clinical specialist degree	4.3%
Ph.D	4.0%
Clinical specialist degree	47.2%
Other continued education	3.4%
Highest education nurses^c^	
Ph.D	1.7%
Master’s degree	14.9%
Other continued education	80.2%
Age^d^	
< 30 years	14.9%
31–40 years	31.6%
41–50 years	23.5%
51–60 years	19.2%
> 60 years	10.9%
Tenure substance abuse and mental health (years)	
Clinical psychologists, mean (SD)	9.98 (9.5)
Nurses, mean (SD)	18.04 (9.2)

^a^20 respondents did not report their gender

^b^After initial cand.psychol degree

^c^After initial Bachelor’s degree

^d^ Three respondents did not report their age

### Acceptability

Among the 418 respondents in the US sample 319 had complete data (76%), but of those with missing data, 49 of the 101 (49%) had missing information on only one item, 24 had 2 to 5 items missing (24%), and the remaining 28 (27%) had more than five items missing. The reporting of unanswered items in the Norwegian sample is based on cases that completed the EBPAS-50 items (*N* = 884). More than three quarters (76.81%) completed all 50 items of the EBPAS-50. No single item was left open by more than 3.3%. Item 17 was omitted most frequently. Moreover, participants provided comments recommending shortening, such as: *“*I think there are too many questions of quite similar nature, which makes it boring and difficult to answer”, or “Too many questions with same content made it tempting to quit without completing”.

### Item reduction

The reduction process was done separately for the US and Norwegian sample. Following the reduction criteria as described in the Statistics section, one item in each of the following subscales having four items were removed: appeal, openness, divergence, monitoring, balance, and burden. The final 36-item version was agreed on following a consensus discussion and is presented in Table 3 for the US and Norwegian samples, respectively. Item 9 from the appeal subscale (“intuitively appealing”) and item 8 from the openness subscale (“Would try therapy/interventions different than usual”) were removed due to content overlap with other items in these scales. Item 34 in the balance subscale (“Satisfied with my skills”) was removed as we considered the content validity of this item as poorer compared to the other three items. Item 3 on the divergence subscale had a low factor loading and was removed. A discrepancy between the US and the Norwegian sample with regard to item 30 (“I prefer to work on my own without oversight”) and item 33 (“I do not need to be monitored”) on the monitoring subscale was solved following a consensus discussion. Consequently, item 33 was removed as it was framed too generally and not specifically referring to work or services. A similar discrepancy occurred with regard to two items on the burden subscale (item 38 “Don’t have time to learn anything new” and item 41 “EBP will cause too much paperwork”). A consensus was reached for excluding item 41, since, unlike the items retained, the referent was not specific to the individual (i.e., EBP will cause too much paperwork *for me*). Also, four items in each of the two subscales with seven items were excluded: limitations and fit. In determining which items to exclude from the limitations subscale, three items were removed on the basis of having the lowest factor loadings. However, item 27 “Families with multiple problems” was excluded despite a high factor loading due to content similarity with item 26 “Clients with multiple problems”. The latter is more universal and was thus retained. There was an additional discrepancy between the US and the Norwegian samples for two items from the limitations subscale (item 24 “Evidence-based practice makes it harder to develop a strong working alliance” and item 28 “Evidence-based practice is not individualized treatment”). Item 28 was retained as it matches the underlying construct of the scale better. For the fit subscale, the three lowest loading items were removed. An additional item was removed (item 19 “Had a say in which evidence-based practice”) as many therapists have less influence on which specific evidence-based practice to adopt rather than how an evidence-based practice will be used. In addition, item 22 “Fit with your treatment philosophy” was excluded despite a high factor loading in the Norwegian sample due to content overlap with item 21 “Fit with your clinical approach”. The English and Norwegian instruments, including scoring instructions, can be found in Additional files 1, 2, 3, and 4.

**Table 3:** EBPAS-50 and EBPAS-36 items standardized factor loadings, means, and standard deviations in US and Norwegian samples

Item no.^a^	Subscales and items	US sample				Norwegian sample			
		EBPAS-50 factor loadings	EBPAS-36 factor loadings	Mean	SD	EBPAS-50 factor loadings	EBPAS-36 factor loadings	Mean	SD
	Scale 1: requirements								
12	Agency required	0.99	0.97	2.65	1.03	0.99	1.00	1.90	1.20
11	Supervisor required	0.88	0.89	2.59	1.05	0.91	0.93	1.83	1.21
13	State required	0.78	0.77	2.72	1.11	0.73	0.79	2.25	1.22
	Scale 2: appeal								
15	Enough training	0.55	0.83	3.13	0.87	0.28	0.68	3.22	0.79
14	Colleagues happy with therapy	0.56	0.71	2.74	0.94	0.34	0.68	2.62	0.89
10	Makes sense	0.89	0.61	3.15	0.81	0.80	0.53	3.04	0.88
9	*Inutitively appealing*	*0.83*	–	*2.91*	*0.89*	*0.75*	–	*2.69*	*1.04*
	Scale 3: openness								
4	Will try therapy/interventions developed by researchers	0.81	0.81	2.79	0.88	0.75	0.68	2.92	0.96
2	Will follow a treatment manual	0.61	0.78	2.64	1.02	0.84	0.86	2.78	1.10
1	Like to use new therapy/interventions	0.62	0.70	2.86	0.91	0.60	0.53	2.80	0.95
8	*Would try therapy/interventions different than usual*	*0.66*	*–*	*2.50*	*0.97*	*0.61*	*–*	*2.29*	*1.09*
	Scale 4: divergence^b^								
7	Would not use manualized therapy/interventions	0.76	0.67	0.82	0.93	0.66	0.76	0.70	1.07
5	Research-based treatments/interventions not useful	0.65	0.59	0.70	0.93	0.55	0.61	0.37	0.72
6	Clinical experience more important	0.42	0.47	2.22	1.00	0.68	0.66	1.76	1.18
3	*Know better than researchers how to care for client*	*0.34*	*–*	*1.66*	*1.02*	*0.56*	*–*	*1.18*	*1.05*
	Scale 5: limitations^b^								
28	Individualized treatment	0.90	0.92	1.35	1.13	0.71	0.80	1.21	1.18
29	Too narrowly focused	0.79	0.89	1.42	1.11	0.77	0.89	1.08	1.05
26	Clients with multiple problems	0.89	0.79	1.15	1.06	0.89	0.74	0.89	1.09
23	*Truly connecting with your clients*	*0.65*	–	*1.29*	*1.08*	*0.74*	–	*0.47*	*0.83*
24	*Develop a strong working alliance*	*0.64*	–	*1.17*	*1.03*	*0.75*	–	*0.49*	*0.81*
25	*Too simplistic*	*0.69*	–	*1.35*	*1.11*	*0.71*	–	*1.02*	*1.07*
27	*Families with multiple problems*	*0.91*	–	*1.29*	*1.15*	*0.88*	–	*0.82*	*0.99*
	Scale 6: fit								
20	Had a say in how I would use the EBP	0.80	0.79	2.89	0.91	0.58	0.67	2.96	1.01
18	Right for your clients	0.78	0.69	3.07	0.92	0.34	0.54	3.42	0.73
21	Fit with your clinical approach	0.65	0.73	2.99	0.94	0.90	0.62	3.12	0.96
16	*Clients wanted it*	*0.72*	*–*	*2.84*	*1.02*	*0.24*	*–*	*2.88*	*0.97*
17	*Knew more about how your clients liked it*	*0.65*	*–*	*3.02*	*0.93*	*0.31*	*–*	*2.55*	*1.04*
19	*Had a say in which EBP*	*0.81*	*–*	*2.83*	*0.98*	*0.49*	*–*	*2.96*	*0.95*
22	*Fit with your treatment philosophy*	*0.58*	*–*	*2.78*	*1.00*	*0.88*	*–*	*3.14*	*0.98*
	Scale 7: monitoring^b^								
31	Looking over my shoulder	0.78	0.88	1.43	1.26	0.73	0.83	0.92	1.22
32	Work does not need to be monitored	0.90	0.85	1.32	1.22	0.87	0.75	0.83	1.13
30	Prefer to work without oversight	0.72	0.71	1.41	1.23	0.69	0.83	0.77	1.10
33	*I do not need to be monitored*	*0.71*	–	*1.24*	*1.26*	*0.88*	–	*0.99*	*1.18*
	Scale 8: balance^b^								
35	Positive outcome is an art	0.60	0.73	1.35	1.20	0.65	0.60	0.84	0.99
36	Therapy is an art and a science	0.73	0.59	2.19	1.37	0.55	0.62	2.20	1.31
37	Overall competence is more important	0.56	0.76	1.23	1.20	0.67	0.61	2.07	1.15
34	*Satisfied with my skills*	0.*77*	*–*	*1.60*	*1.35*	*0.19*	–	*2.09*	*1.03*
	Scale 9: burden^b^								
39	Can΄t meet other obligations	0.72	0.81	0.95	1.10	0.60	0.70	1.26	1.14
40	How to fit evidence-based practice in	0.71	0.67	1.24	1.13	0.83	0.61	0.85	1.03
38	Don΄t have time to learn anything new	0.51	0.57	0.65	0.96	0.60	0.76	0.77	1.04
41	*Cause too much paperwork*	*0.63*	–	*1.22*	*1.04*	*0.54*	–	*0.83*	*0.99*
	Scale 10: job security								
43	Help me get a new job	0.89	0.98	1.94	1.25	0.99	0.95	1.47	1.30
42	Help me keep my job	0.81	0.80	1.69	1.32	0.62	0.60	0.83	1.16
44	Make it easier to find work	0.61	0.61	1.75	1.30	0.89	0.91	1.48	1.27
	Scale 11: organizational support								
46	Training provided	0.81	0.86	3.12	0.87	0.92	0.92	2.69	1.19
47	Ongoing support provided	0.68	0.82	3.24	0.80	0.87	0.87	2.44	1.21
45	Continuing education credits provided	0.75	0.74	2.89	1.04	0.67	0.61	1.75	1.35
	Scale 12: feedback								
49	Feedback helps me to be better	0.74	0.83	3.23	0.82	0.93	0.96	3.56	0.81
50	Supervision helps me to be better	0.68	0.78	3.27	0.83	0.63	0.72	3.58	0.81
48	Enjoy feedback on performance	0.62	0.69	3.12	0.88	0.82	0.84	3.38	0.86

All factor loadings are standardized. Italicized items are items removed from the EBPAS-36

^a^Item number from original EBPAS-50

^b^Reversed scale

### Subscale score correlations

The correlation coefficients (Pearson *r*) between the 12 EBPAS-36 subscales are presented in Table 4 separately for the Norwegian and US samples. Norwegian sample: the highest correlations were between the limitations and the divergence subscales (*r* = .56), the job Security and the organizational support subscales (*r* = .53), the openness and the divergence subscales (*r* = −.49), the appeal and the fit subscales (*r* = .41), the organizational support and the openness subscales (*r* = .40), the divergence and the balance subscales (*r* = .38), and the limitations and openness subscales (*r* = −.37), all in the expected directions. There were some similarities with the Norwegian sample correlations that emerged in the US sample, specifically between organizational support and openness (*r* = .40). Though the highest correlation found was between appeal and organizational support (*r* = .57).

**Table 4:** EBPAS-36 scale factor intercorrelations for US and Norwegian samples

Subscales	1	2	3	4	5	6	7	8	9	10	11	12
1. Requirements	–	.41^**^	.25^**^	−.14^**^	.−17^**^	.16^**^	−.10^*^	−.08	−.16^**^	.18^**^	.34^**^	.17^**^
2. Appeal	.36^**^	–	.38^**^	−.18^**^	−.06	.38^**^	−.12^*^	.00	.03	.21^**^	.57^**^	.35^**^
3. Openness	.25^**^	.31^**^	–	−.14^**^	−.09	.31^**^	−.10	−.02	−.02	.32^**^	.40^**^	.31^**^
4. Divergence	−.33^**^	−.14^**^	−.49^**^	–	.31^**^	−.03	.30^**^	.27^**^	.22^**^	−.07	−.09	−.07
5. Limitations	−.20^**^	.04	−.37^**^	.56^**^	–	.00	.33^**^	.29^**^	.34^**^	−.11^*^	−.10^*^	−.08
6. Fit	.06	.41^**^	.15^**^	.06	.06	–	.07	.18^**^	.11^**^	.27^**^	.34^**^	.29^**^
7. Monitoring	−.18^**^	−.03	−.23^**^	.36^**^	.32^**^	.04	–	.28^**^	.27^**^	−.05	−.07	−.26^**^
8. Balance	−.18^**^	.11^**^	−.13^**^	.38^**^	.32^**^	.22^**^	.30^**^	–	.17^**^	−.06	−.09	−.07
9. Burden	.02	.12^**^	−.10^**^	.15^**^	.22^**^	.00	.16^**^	.13^**^	–	.02	.09	−.11^*^
10. Job security	.22^**^	.12^**^	.29^**^	−.24^**^	−.13^**^	.06	−.10^**^	−.02	.02	–	.34^**^	.15^**^
11. Organizational support	.28^**^	.30^**^	.40^**^	−.28^**^	−.15^**^	.17^**^	−.15^**^	−.01	.10^**^	.53^**^	–	.31^**^
12. Feedback	.13^**^	.24^**^	.30^**^	−.10^**^	−.11^**^	.22^**^	−.23^**^	.09^*^	.01	.18^**^	.37^**^	–

The coefficients for the US sample (*N* = 418) are in the upper diagonal, and for the Norwegian sample (*N* = 838) in the lower diagonal. ^*^ indicates significance at the *p* < .05 level, ^**^ indicates significance at the *p* < .001 level

### Confirmatory factor analyses

In the US sample, after adjusting for the nested data structure (i.e., providers within programs), the absolute fit was significant (χ^2^ = 968.85, *p* < .001); however, model fit was adequate in terms of low misspecification (RMSEA = .045, CI 90% [.040, .049]; SRMR = .05), and fair with regard to incremental fit (CFI = .93, TLI = .91). The standardized factor loadings ranged from .48–.98 (all *p’s* < .001). In the Norwegian sample, the absolute fit again was significant fit (χ^2^ = 1125.04, *p* < .001), but adequate in terms of low misspecification (RMSEA = .052, CI 90% [.047, .056]; SRMR = .07), and fair in terms of incremental fit (CFI = .91, TLI = .89). The standardized factor loadings ranged from .52 to 1.00 (all *p’s* < .001, except for item 18 with *p* = .003).

### Internal consistency

The internal consistency of the US and Norwegian EBPAS-36, as well as the US EBPAS-50 [11, 13], is presented in Table 5. The US EBPAS-36 total scale Cronbach’s α was .80, with subscale α that ranged between .60 and .91. The divergence subscale had the lowest α (.60), which could not be improved by removing the lowest item-total correlation, and hence could be interpreted as questionable [31]. The remaining subscales had Cronbach’s α above .70, indicating acceptable to excellent levels of internal consistency. The Norwegian EBPAS-36 total scale had Cronbach’s α .86, and subscales α ranging between .61 and .92. The Appeal, Fit, Balance and Divergence subscales had the lowest alphas (.61, .62, .64 and .68, respectively) and did not improve above > .70 following removal of items with low item-total correlations, which was less satisfactory. The remaining subscales α were above > .70. Compared to the Cronbach’s α in the US EBPAS-50, both the US and Norwegian EBPAS-36 had lower α values, as expected for shorter scales, as well as implementation constructs of a broad nature.

**Table 5:** Internal consistency, Cronbach’s α, for US and Norwegian samples, and original version of EPBPAS-50

Domain	Cronbach’s α		
	US EBPAS-36	Norwegian EBPAS-36	Original version EBPAS-50^a^
Requirements	.91	.92	.90
Appeal	.75	.61	.80
Openness	.81	.76	.78
Divergence	.60	.68	.59
Limitations	.90	.85	.92
Fit	.77	.62	.88
Monitoring	.85	.84	.87
Balance	.74	.64	.79
Burden	.76	.74	.77
Job security	.82	.86	.82
Organizational support	.84	.84	.85
Feedback	.80	.85	.82
Total scale	.79	.86	

^a^Reported by [11] and [13]; discrepancies between Cronbach’s alphas from the original EBPAS-50 and US EBPAS-36 on those subscales where no items were removed are due to the alphas in the original EBPAS-50 being conducted on an data set in which missing values on items were imputed

## Discussion

There is a profound need for shorter and pragmatic instruments that at the same time cover a wide spectrum of measurement constructs in implementation research. This article describes the shortening of the EBPAS-50 from 50 to 36 items based on data collected in the US and in Norway. The revised instrument, named EBBAS-36, measures 12 dimensions of provider’s attitudes to adopt new practices in mental health care service settings, similarly as the original EBPAS-50 does. Data from both cultures indicated adequate psychometric properties of the EBPAS-36, hence being cross-culturally valid. The shortened version is not compromised by narrowing of the measurement domain as it retains the original 12 factor structure. The internal consistency of most subscales was good to high, and on par with the EBPAS-50. Some subscales had slightly lower internal consistencies in the US and the Norwegian versions: these were the appeal, the fit, and the balance subscales. Lower internal consistency is in general expected if reducing the number of items. Given that the EBPAS-36 contains only three items per factor, the lowered internal consistency may be considered as adequate, especially if compensated for by increasing the sample size in research employing these short versions. Another factor in the results may be the complexity of the measured concepts, as more heterogeneous constructs also can attenuate alpha where there are lower item-total correlations. Since attitudes towards implementation of EBP may be considered as relatively broad in scope, we consider the slightly reduced reliability to be well compensated by the broader validity of retaining the original 12 dimensions and the practicality of a brief measure that can be used more efficiently for research, organizational development, and provider development. Furthermore, several comments to the 50-item version indicated that responders were annoyed or fatigued by having to answer several seemingly identical items. Shortening the instrument is rather more likely to strengthen the validity of the scale due to fewer response biases related to irritated or fatigued respondents, which in the test literature should decrease the phenomenon of “satisficing” [32].

The literature has suggested multiple criteria for “pragmatic” measures including being important to stakeholders; having low burden; being sensitive to change; being broadly applicable; can be used for benchmarking; has norms; is unlikely to cause harm; is psychometrically strong; and is related to theory or model [8, 9]. This revision of the EBPAS-50 to a more brief and focused measure fits most of these criteria when considered in the context of studies utilizing the EBPAS and EBPAS-50, from which the EBPAS-36 was adapted. First, the EBPAS and EBPAS-50 may be deemed to be important to stakeholders by virtue of the wide use of the measures for research, service improvement, and practice [33]. Second, burden for the measure is low for respondents and the measure can be completed in just a few minutes. Third, the EBPAS and/or EBPAS-50 have been used in a variety of settings including health/medicine [34], mental health [35], substance abuse [36], education [37], social care [38], and across countries and cultures [34, 39, 40]. Fourth, norms have been established for behavioral health settings in the United States [21], and thus can be used for benchmarking in this setting, and as the measures are utilized more broadly, evidence and normative data will become available to aid in interpretation and in understanding of both mean scores and variability in responses across countries, cultures, and various health settings. Fifth, the measure is very unlikely to cause harm. Sixth, the measure is psychometrically strong [21]. And seventh, the measure is clearly related to theory including theories of links between attitudes and behaviors [41] and as identified in multiple implementation frameworks [10, 14, 42]. While further testing of the EBPAS-36 is clearly warranted, the present study conducted across cultures, languages, and using rigorous approaches to factor structures, reliability, and validity—along with consideration of previous work—supports this new measure as both brief and having very high potential for being pragmatic.

Some limitations of this study should be mentioned. One concerns the low response rate for the Norwegian sample. Some of the Norwegian respondents provided written comments to the survey related to the response scale. The Norwegian translation used the same anchor points as the original version: 0-not at all, 1-to a slight extent, 2-to a moderate extent, 3-to a great extent and 4-to a very great extent. During piloting of the translation, a different response scale was examined, which led to response problems related to two of the items containing negations. In order to avoid changing both the phrasing of the items and the scaling, the original response scale was used. Another concern may be the introductory cross-cultural definition differences that were used to describe the basis for evidence. Normally, comparability across cultures is the rule, which we bypassed for good reasons. The English version describes “evidence-based practice” as referring to any interventions that is supported by empirical research. However, the Norwegian version limited the evidence-based definitions to the methods used (i.e., therapies, interventions). This was an attempt to distinguish the more comprehensive concept of *evidence-based practice* (referring to the integration of the best available research with clinical expertise in the context of patient characteristics, culture, and preferences) from the concept of *evidence-based treatment*, which refers to special treatments supported by empirical research. Written comments from the Norwegian respondents indicated some had difficulties with discerning these two complex concepts. Future use of the instrument should pay special attention to this challenge, for example, by comparing the outcome of using these two different introductory statements (broad versus narrow definition) by randomly distributing them to two different samples. However, the solutions in Norwegian and US were so similar, that the impact of the difference in definitions, if existent, is probably minimal.

EBPAS-50 was originally developed both for research and for applied purposes. The intention behind developing the measure was in large extent to provide a relatively brief measure, both to be used in studies of organizational and individual readiness to implement new evidence-based interventions, and for understanding factors related to adoption, implementation, and continued use of evidence-based interventions [20]. The presented 36-item version builds upon this intention, providing an even shorter instrument measuring the same dimensions as the EBPAS-50. Our procedure for creating a short and pragmatic version may serve as a model for other researchers within the field. Future research on the EBPAS-36 may examine how organizational and individual factors relate to the various EBPAS-36 attitude dimensions, which may help tailor implementation strategies that promote an organizational climate that adopt new interventions positively.

## Conclusions

The EBPAS-36 has adequate psychometric properties both in US and in Norwegian samples, hence, indicating cross-cultural validity. It is a brief, pragmatic, and more user-friendly instrument than the EPBAS-50, yet maintains a broad scope by retaining the original 12 measurement domains.

## Additional files

Additional file 1: The Evidence-based Practice Attitude Scale-36 (EBPAS-36), English version PDF. (PDF 96 kb)

Additional file 2: The Evidence-based Practice Attitude Scale-36 (EBPAS-36), Norwegian version PDF. (PDF 212 kb)

Additional file 3: The Evidence-based Practice Attitude Scale-36 (EBPAS-36), English version Scoring instructions PDF. (PDF 217 kb)

Additional file 4: The Evidence-based Practice Attitude Scale-36 (EBPAS-36), Norwegian version Scoring instructions PDF. (PDF 108 kb)

## Abbreviations

CFA: Confirmatory factor analysis
EBP: Evidence-based practice
EBPAS: Evidence-based Practice Attitude Scale
EPIS: Exploration Preparation Implementation and Sustainment
