# Supplementary material for: The Evidence-based Practice Attitude Scale-36 (EBPAS-36): a brief and pragmatic measure of attitudes to evidence-based practice validated in US and Norwegian samples
Source: Implement Sci. 2017 Apr 4;12:44. doi: 10.1186/s13012-017-0573-0 (PMC5379724; doi:10.1186/s13012-017-0573-0)
Supplement: Supplementary file 1 — The Evidence-based Practice Attitude Scale-36 (EBPAS-36), English version PDF. (PDF 96 kb) [file 13012_2017_573_MOESM1_ESM.pdf]

# Evidence-Based Practice Attitude Scale (EBPAS)© 36

Gregory A. Aarons
gaarons@ucsd.edu

The EBPAS assesses mental health provider attitudes toward adoption of innovation and evidence-based practices (EBPs) in mental health and social service settings. Items are presented on a 5-point Likert scale ranging from 0 "Not at All" to 4 "To a Very Great Extent".

**Reference**

Rye, M., Torres, E. M., Friborg, O., Skre, I., & Aarons, G. A. (under review). The Evidence-based Practice Attitude Scale-36 (EPBAS-36): A brief and pragmatic measure of attitudes to evidence-based practice validated in Norwegian and U.S. samples. *Implementation Science*.

For information contact Gregory Aarons: gaarons@ucsd.edu

# Evidence-Based Practice Attitude Scale (EBPAS)© 36

The following questions ask about your feelings about using new types of therapy, interventions, or treatments. Manualized therapy refers to any intervention that has specific guidelines and/or components that are outlined in a manual and/or that are to be followed in a structured/ predetermined way.

Please indicate the extent to which you agree with each item using the below scale.

| 0 | 1 | 2 | 3 | 4 |
|---|---|---|---|---|
| **Not at all** | **Slight extent** | **Moderate extent** | **Great extent** | **Very great extent** |

*For questions 1-6: Circle the number indicating the extent to which you agree with each item using the following scale:*

1. I like to use new types of therapy/interventions to help my clients ............................................ 0 1 2 3 4

2. I am willing to try new types of therapy/interventions even if I have to follow a treatment manual ............................................................................................................ ..... 0 1 2 3 4

3. I am willing to use new and different types of therapy/interventions developed by researchers ........................................................................................................................... 0 1 2 3 4

4. Research based treatments/interventions are not clinically useful .............................................. 0 1 2 3 4

5. Clinical experience is more important than using manualized therapy/treatment ....................... 0 1 2 3 4

6. I would not use manualized therapy/interventions ...................................................................... 0 1 2 3 4

*For questions 6-12: If you received training in a therapy or intervention that was new to you, how likely would you be to adopt it if:*

7. it “made sense” to you? ............................................................................................................ 0 1 2 3 4

8. it was required by your supervisor? .......................................................................................... 0 1 2 3 4

9. it was required by your agency? ............................................................................................... 0 1 2 3 4

10. it was required by your state? ................................................................................................. 0 1 2 3 4

11. it was being used by colleagues who were happy with it? ....................................................... 0 1 2 3 4

12. you felt you had enough training to use it correctly? .............................................................. 0 1 2 3 4

| 0<br>Not at all | 1<br>Slight extent | 2<br>Moderate extent | 3<br>Great extent | 4<br>Very great extent |
|---|---|---|---|---|

*For questions 13-15: If you received training in a therapy or intervention that was new to you, how likely would you be to adopt it if:*

13. you knew it was right for your clients ........................................................................ 0 1 2 3 4

14. you had a say in how you would use the evidence-based practice ........................................... 0 1 2 3 4

15. it fit with your clinical approach ............................................................................... 0 1 2 3 4

*For questions 16-36: Circle the number indicating the extent to which you agree with each item using the following scale:*

16. Evidence-based practice is not useful for clients with multiple problems ................................ 0 1 2 3 4

17. Evidence-based practice is not individualized treatment ......................................................... 0 1 2 3 4

18. Evidence-based practice is too narrowly focused .................................................................. 0 1 2 3 4

19. I prefer to work on my own without oversight... ..................................................................... 0 1 2 3 4

20. I do not want anyone looking over my shoulder while I provide services ............................... 0 1 2 3 4

21. My work does not need to be monitored. .................................................................................... 0 1 2 3 4

22. A positive outcome in therapy is an art more than a science .................................................... 0 1 2 3 4

23. Therapy is both an art and a science ........................................................................................... 0 1 2 3 4

24. My overall competence as a therapist is more important than a particular approach ................ 0 1 2 3 4

25. I don't have time to learn anything new ...................................................................................... 0 1 2 3 4

26. I can't meet my other obligations ................................................................................................. 0 1 2 3 4

27. I don't know how to fit evidence-based practice into my administrative work ........................ 0 1 2 3 4

28. Learning an evidence-based practice will help me keep my job ............................................. 0 1 2 3 4

29. Learning an evidence-based practice will help me get a new job ............................................ 0 1 2 3 4

30. Learning an evidence-based practice will make it easier to find work .................................... 0 1 2 3 4

31. I would learn an evidence-based practice if continuing education credits were provided.......................................................................................................................... 0 1 2 3 4

| 0 | 1 | 2 | 3 | 4 |
|---|---|---|---|---|
| **Not at all** | **Slight extent** | **Moderate extent** | **Great extent** | **Very great extent** |

32. I would learn an evidence-based practice if training were provided ........................................ 0 1 2 3 4

33. I would learn an evidence-based practice if ongoing support was provided ............................ 0 1 2 3 4

34. I enjoy getting feedback on my job performance ..................................................................... 0 1 2 3 4

35. Getting feedback helps me to be a better therapist/case manager ............................................ 0 1 2 3 4

36. Getting supervision helps me to be a better therapist/case manager ........................................ 0 1 2 3 4
