# Supplementary material for: The Evidence-based Practice Attitude Scale-36 (EBPAS-36): a brief and pragmatic measure of attitudes to evidence-based practice validated in US and Norwegian samples
Source: Implement Sci. 2017 Apr 4;12:44. doi: 10.1186/s13012-017-0573-0 (PMC5379724; doi:10.1186/s13012-017-0573-0)
Supplement: Supplementary file 2 — The Evidence-based Practice Attitude Scale-36 (EBPAS-36), Norwegian version PDF. (PDF 212 kb) [file 13012_2017_573_MOESM2_ESM.pdf]

Evidence-Based Practice Attitude Scale (EBPAS)© 36

Gregory A. Aarons
gaarons@ucsd.edu

Norsk oversettelse

EBPAS måler helsepersonell sin innstilling til å ta i bruk nye terapier, intervensjoner eller behandlinger. Hvert spørsmål besvares på en Likert-skala fra 0 "Helt uenig" til 4 "Helt enig".

Referanse

Rye, M., Torres, E. M., Friborg, O., Skre, I., & Aarons, G. A. (under review). The Evidence-based Practice Attitude Scale-36 (EPBAS-36): A brief and pragmatic measure of attitudes to evidence-based practice validated in Norwegian and U.S. samples. *Implementation Science*.

For informasjon, kontakt:
Gregory Aarons: gaarons@ucsd.edu
Marte Rye: marte.rye@uit.no, marte.rye@unn.no

Evidence-Based Practice Attitude Scale (EBPAS)© 36

De følgende spørsmål omhandler din innstilling til å ta i bruk nye terapier, intervensjoner eller behandlinger. Manualbasert terapi refererer til enhver intervensjon som har spesifikke retningslinjer og/eller komponenter som er beskrevet i en manual og/eller som skal følges på en strukturert/forutbestemt måte. Evidensbaserte metoder refererer til enhver intervensjon som støttes av empirisk forskning.

Vennligst sett en ring rundt tallet som viser i hvilken grad du er enig med hver påstand ved hjelp av følgende skala:

0	1	2	3	4
**Helt uenig**	**Litt enig**	**Moderat enig**	**Ganske enig**	**Helt enig**

For spørsmål 1-6: Sett en ring rundt tallet som viser i hvilken grad du er enig med hver påstand ved hjelp av skalaen ovenfor:

1. Jeg liker å bruke nye former for terapi/intervensjoner for å hjelpe mine pasienter……….... 0 1 2 3 4
2. Jeg er villig til å prøve nye former for terapi/intervensjoner selv om jeg da må følge en behandlingsmanual.. 0 1 2 3 4
3. Jeg er villig til å bruke nye og forskjellige former for terapi/intervensjoner som er utviklet av forskere…... 0 1 2 3 4
4. Forskningsbaserte behandlingsformer/intervensjoner er ikke klinisk nyttige……………... 0 1 2 3 4
5. Klinisk erfaring er viktigere enn bruk av manualbasert terapi/behandling……..................... 0 1 2 3 4
6. Jeg ville ikke brukt manualbaserte terapier/intervensjoner.. 0 1 2 3 4

For spørsmål 7-15: Hvis du fikk opplæring i en terapi eller intervensjon som var ny for deg, hvor sannsynlig er det at du ville ta den i bruk gitt at:

7. den virket fornuftig?.. 0 1 2 3 4
8. det ble pålagt av din leder?... 0 1 2 3 4
9. det ble pålagt av din arbeidsplass? 0 1 2 3 4
10. det ble pålagt av myndighetene?... .... 0 1 2 3 4
11. den ble brukt av kollegaer som var fornøyd med den?.. 0 1 2 3 4
12. du følte du hadde nok opplæring til å bruke den riktig?.......... .... 0 1 2 3 4
13. du visste den var velegnet for dine pasienter……………... 0 1 2 3 4
14. du hadde innflytelse på hvordan du skulle bruke den evidensbaserte metoden................... 0 1 2 3 4
15. den passet med din kliniske tilnærming.. 0 1 2 3 4

Evidence-Based Practice Attitude Scale (EBPAS)© 36

0	1	2	3	4
**Helt uenig**	**Litt enig**	**Moderat enig**	**Ganske enig**	**Helt enig**

For spørsmål 16-36: Sett en ring rundt tallet som viser i hvilken grad du er enig med hver påstand ved hjelp av skalaen ovenfor:

16. Evidensbaserte metoder er ikke nyttige for pasienter med sammensatte problemer.. 0 1 2 3 4
17. Evidensbaserte metoder er ikke tilpasset den enkelte pasient.. 0 1 2 3 4
18. Evidensbasert praksis har for snevert fokus 0 1 2 3 4
19. Jeg foretrekker å jobbe på egen hånd uten tilsyn... 0 1 2 3 4
20. Jeg ønsker ikke at noen kikker over skulderen min mens jeg gjør jobben min.............. 0 1 2 3 4
21. Det er unødvendig å holde øye med arbeidet mitt... 0 1 2 3 4
22. Et positivt utfall i terapi er følge av kunst mer enn en vitenskap 0 1 2 3 4
23. Terapi er både kunst og vitenskap…….. 0 1 2 3 4
24. Min terapeutiske kompetanse er viktigere enn en bestemt tilnærming................................ 0 1 2 3 4
25. Jeg har ikke tid til å lære noe nytt 0 1 2 3 4
26. Jeg kan ikke oppfylle mine andre plikter... 0 1 2 3 4
27. Jeg vet ikke hvordan jeg skal få passet inn evidensbaserte metoder i mine administrative oppgaver.. 0 1 2 3 4
28. Å lære en evidensbasert metode vil hjelpe meg å beholde jobben min................................ 0 1 2 3 4
29. Å lære en evidensbasert metode vil hjelpe meg med å få en ny jobb................................... 0 1 2 3 4
30. Å lære en evidensbasert metode vil gjøre det lettere å finne arbeid 0 1 2 3 4
31. Jeg vil lære en evidensbasert metode hvis det gir godkjente studiepoeng som etter-/ videreutdanning 0 1 2 3 4
32. Jeg ville lære en evidensbasert metode hvis det ble gitt opplæring…..……....................... 0 1 2 3 4
33. Jeg ville lære en evidensbasert metode hvis det ble gitt kontinuerlig oppfølging................ 0 1 2 3 4
34. Jeg liker å få tilbakemelding på jobben jeg gjør 0 1 2 3 4
35. Å motta tilbakemelding hjelper meg å bli en bedre terapeut.. 0 1 2 3 4
36. Å motta veiledning hjelper meg til å bli en bedre terapeut 0 1 2 3 4
