# Supplementary material for: The Evidence-based Practice Attitude Scale-36 (EBPAS-36): a brief and pragmatic measure of attitudes to evidence-based practice validated in US and Norwegian samples
Source: Implement Sci. 2017 Apr 4;12:44. doi: 10.1186/s13012-017-0573-0 (PMC5379724; doi:10.1186/s13012-017-0573-0)
Supplement: Supplementary file 3 — The Evidence-based Practice Attitude Scale-36 (EBPAS-36), English version Scoring instructions PDF. (PDF 217 kb) [file 13012_2017_573_MOESM3_ESM.pdf]

EBPAS-36 (©Gregory A. Aarons, Ph.D.)
Evidence-Based Practice Attitude Scale
Items, Factor Loadings, Chronbach's Alphas, and Scoring

Reference:

Rye, M., Torres, E. M., Friborg, O., Skre, I., & Aarons, G. A. (under review). The Evidence-based Practice Attitude Scale-36 (EPBAS-36): A brief and pragmatic measure of attitudes to evidence-based practice validated in Norwegian and U.S. samples. *Implementation Science*.

Item #	Scale	Factor Loading	Alpha
	**Scale 1: Requirements**		.91
8	Supervisor required	.89	
9	Agency required	.97	
10	State required	.77	
	**Scale 2: Appeal**		.75
7	Makes sense	.61	
11	Colleagues happy with therapy	.71	
12	Enough training	.83	
	**Scale 3: Openness**		.81
2	Will follow a treatment manual	.78	
3	Will try therapy/interventions developed by researchers	.81	
1	Like to use new therapy/interventions	.70	
	**Scale 4: Divergence**		.60
4	Research based treatments/interventions not useful	.59	
6	Would not use manualized therapy/interventions	.67	
5	Clinical experience more important	.47	
	**Scale 5: Limitations**		.90
16	Clients with multiple problems	.79	
17	Not individualized treatment	.92	
18	Too narrowly focused	.89	
	**Scale 6: Fit**		.77
13	Right for your clients	.69	
14	Had a say in how to use the evidence-based practice	.79	
15	Fit with your clinical approach	.73	
	**Scale 7: Monitoring**		.85
19	Work without oversight	.71	
20	Looking over my shoulder	.88	
21	My work does not need to be monitored	.85	

	**Scale 8: Balance**		.74
22	Positive outcome is an art	.73	
23	Therapy is an art and a science	.59	
24	Overall competence is more important	.76	
	**Scale 9: Burden**		.76
25	Don’t have time to learn anything new	.57	
26	Can’t meet other obligations	.81	
27	How to fit evidence-based practice in	.67	
	**Scale 10: Job security**		.82
28	Help me keep my job	.80	
29	Help me get a new job	.98	
30	Make it easier to find work	.61	
	**Scale 11: Organizational Support**		.84
31	Continuing education credits provided	.74	
32	Training provided	.86	
33	Ongoing support provided	.82	
	**Scale 12: Feedback**		.80
34	Enjoy feedback on performance	.69	
35	Feedback helps me to be better	.83	
36	Supervision helps me to be better	.78	
	**EBPAS-36 Total Scale**		.79

SCORING THE SCALES

The score for each subscale is created by computing a mean score for each set of items that load on a given subscale. For example, items 8, 9, and 10 constitute Requirements subscale. If there is missing data in your data set, computing means may be done allowing for one fewer items than make up the scale.

COMPUTING THE TOTAL SCORE

Only for the total score (not the individual scale scores), items from subscale 4 (Divergence), subscale 5 (Limitations), subscale 7 (Monitoring), subscale 8 (Balance) and subscale 9 (Burden) **must be reverse scored** and the subscale score recomputed. After the reverse scoring is complete, then a mean of the scale scores may be computed to yield the mean score for the total EBPAS-36 Item Score.

You may contact Dr. Aarons by email at: gaarons@ucsd.edu
