# Supplementary material for: The Evidence-based Practice Attitude Scale-36 (EBPAS-36): a brief and pragmatic measure of attitudes to evidence-based practice validated in US and Norwegian samples
Source: Implement Sci. 2017 Apr 4;12:44. doi: 10.1186/s13012-017-0573-0 (PMC5379724; doi:10.1186/s13012-017-0573-0)
Supplement: Supplementary file 4 — The Evidence-based Practice Attitude Scale-36 (EBPAS-36), Norwegian version Scoring instructions PDF. (PDF 108 kb) [file 13012_2017_573_MOESM4_ESM.pdf]

EBPAS-36 (©Gregory A. Aarons, Ph.D.) Evidence-Based Practice Attitude Scale

Norsk versjon

Referanse:

Rye, M., Torres, E. M., Friborg, O., Skre, I., & Aarons, G. A. (under review). The Evidence-based Practice Attitude Scale-36 (EPBAS-36): A brief and pragmatic measure of attitudes to evidence-based practice validated in Norwegian and U.S. samples. *Implementation Science*.

Spørsmål #	Skala	Faktorladning	Alpha
	**Skala 1: Requirements**		.92
8	Pålagt fra leder	.93	
9	Pålagt fra arbeidsplass	1.00	
10	Pålagt fra myndigheter	.79	
	**Skala 2: Appeal**		.61
7	Virket fornuftig	.53	
11	Kollegaer fornøyd	.68	
12	Nok opplæring	.68	
	**Skala 3: Openness**		.76
2	Vil følge en behandlingsmanual	.86	
3	Vil prøve terapier/intervensjoner utviklet av forskere	.68	
1	Liker ta i bruk nye terapier/intervensjoner	.53	
	**Skala 4: Divergence**		.68
4	Forskningsbaserte ikke klinisk nyttige	.61	
6	Ønsker ikke bruke manualiserte terapier/intervensjoner	.76	
5	Klinisk erfaring viktigere	.66	
	**Skala 5: Limitations**		.85
16	Sammensatte problem	.74	
17	Ikke tilpasset pasient	.80	
18	Snevert fokus	.89	
	**Skala 6: Fit**		.62
13	Velegnet for pasient	.54	
14	Hvordan bruke metode	.67	
15	Passet med klinisk tilnærming	.62	
	**Skala 7: Monitoring**		0.84
19	Foretrekke jobbe uten tilsyn	.83	
20	Kikke over skulderen	.83	
21	Holde øye med arbeidet	.75	
	**Skala 8: Balanse**		.64
22	Positivt utfall er kunst	.60	
23	Terapi både kunst og vitenskap	.62	
24	Terapeutisk kompetanse er viktigere	.61	

Spørsmål #	Skala	Faktorladning	Alpha
	**Skala 9: Burden**		.74
25	Ikke tid lære nytt	.76	
26	Klarer ikke oppfylle forpliktelser	.70	
27	Hvordan passe inn	.61	
	**Skala 10: Job security**		.86
28	Hjelpe meg beholde jobben	.60	
29	Hjelpe meg få ny jobb	.95	
30	Lettere finne arbeid	.91	
	**Skala 11: Organizational support**		.84
31	Godkjent videreutdanning	.61	
32	Opplæring	.92	
33	Kontinuerlig oppfølging	.87	
	**Skala 12: Feedback**		.85
34	Liker tilbakemelding	.84	
35	Tilbakemelding hjelper bli bedre	.96	
36	Veiledning hjelper bli bedre	.72	
	**Epbas-36 total skåre**		**.86**

SCORING THE SCALES

The score for each subscale is created by computing a mean score for each set of items that load on a given subscale. For example, items 1, 2, and 3 constitute Requirements subscale. If there is missing data in your data set, computing means may be done allowing for one fewer items than make up the scale.

COMPUTING THE TOTAL SCORE

Only for the total score (not the individual scale scores), items from Divergence, Limitations, Monitoring, Competence and Burden subscales **must be reverse scored** and the subscale score recomputed. After the reverse scoring is complete, then a mean of the scale scores may be computed to yield the mean score for the total EBPAS-36 Item Score.

You may contact Dr. Gregory Aarons by email at: gaarons@ucsd.edu
